# Using operational research as a tool to improve eye health services and systems in low-and middle-income settings: lessons from India and Nepal

**DOI:** 10.1186/s12909-025-07803-6

**Published:** 2025-08-26

**Authors:** Ruchi Priya, Kenneth L. Bassett, Gudlavalleti Venkata Satyanarayana Murthy, Kieran S. O’Brien, Katie Judson, Suzanne S. Gilbert, Sirshendu Chaudhuri, Varun Agiwal

**Affiliations:** 1 Pragyaan Sustainable Health Outcomes Foundation, Level 2, Kapil Kavuri HUB, Financial District, Gachibowli Hyderabad- 500032, Hyderabad, Telangana India; 2 Indian Institute of Public Health, Hyderabad, India; 3 London School of Hygiene and Tropical Medicine, London, UK; 4 Seva Canada, Vancouver, Canada; 5 Seva Foundation, Berkeley, CA USA; 6 University of British Columbia, Vancouver, Canada; 7 Francis I. Proctor Foundation, University of California, San Francisco (UCSF), USA

**Keywords:** Operational research, Capacity building, Eye care, Mentorship, Low-and Middle-Income countries

## Abstract

**Background:**

Operational Research (OR), as part of a quality assurance program, has become a standard feature of most health institutions in most high-income countries. In contrast, in low-income settings, operational research is less common, and almost no one has asssed operational research capacity building (ORCB) as a tool to improve efficacy, efficiency and quality in these settings. This study evaluated the impact of an ORCB program on participants’ research competencies and the extent to which research findings were implemented in practice.

**Materials and methods:**

This study combined quantitative and qualitative data to evaluate an ORCB intervention in eye hospitals in Nepal (3 sites) and northern India (1 site) from 2019 to 2022. A self-reported questionnaire was administered at the end of the study period, and formal interviews were conducted. The questionnaire covered knowledge improvement, practice implementation, and motivating and challenging factors. Statistical analysis included paired t-tests to compare pre- and post-training scores. Qualitative data were gathered through interviews and observations and analysed thematically.

**Results:**

The program demonstrated significant improvements in participants’ research knowledge gain. Quantitative analysis revealed substantial gains in knowledge (*p*-values < 0.05 for all domains). Post-training, 66.7% developed study protocols, and 60% trained other staff or students. Qualitative feedback indicated overall positive impacts, including enhanced research and operational activities. However, reported challenges such as inconsistent mentorship quality, poor internet connectivity during online sessions, and difficulty in balancing clinical work with research. Despite these challenges, there was notable improvement in research practice and internal training within hospitals, and the program’s approach was appreciated for its effectiveness.

**Conclusion:**

The study highlights the need for standardized training modules, consistent mentorship, and stronger institutional support. Building operational research capacity in resource-poor settings with limited administrative staff and weak data infrastructure improves individual staff knowledge and skills. Participants learned about scientific principles of reliability and validity and their importance to efforts to improve service equity, efficiency, and effectiveness.

**Supplementary Information:**

The online version contains supplementary material available at 10.1186/s12909-025-07803-6.

## Background

OR in healthcare is essential for improving the efficiency, effectiveness, and equity of health services and systems [1–3]. OR is crucial for addressing a wide range of operational challenges by building on existing monitoring and evaluation infrastructure, including routine administrative data and quality assurance programs [1–4]. It focuses on developing solutions to current operational problems, ensuring that decisions are based on solid evidence. This data-driven approach leads to more informed and effective outcomes [1–4]. 

In the context of eye care, OR can significantly enhance patient outcomes by optimizing various aspects of service delivery and resource utilization. For example, OR can help in developing decision support tools for automated patient scheduling based on factors like patient risk, referral-to-treatment times, and clinic capacities [1–7]. This prioritizes patients efficiently and reduces waiting times [1–8]. Additionally, OR can identify bottlenecks and improve the efficiency of eye care service delivery, such as increasing the follow-up rates etc [1–3]. By enhancing the quality of service delivery through data analysis and systematic use of program data, eye care services can become more effective and patient-centred [1–4]. Increasing the coverage and reach of eye care services at the population level is another critical area where OR proves invaluable [1–4]. It can identify underserved areas and optimize resource allocation to ensure affordable access to eye care. Evaluating the impact of interventions, like community-based eye health education programs, on service uptake and patient outcomes, further underscores the importance of OR in eye care services [1–7]. 

The application of OR varies significantly between high-income countries (HICs) and low- and middle-income countries (LMICs), reflecting differences in healthcare infrastructure, resource availability, and training needs [1, 7, 8]. In high-income countries, OR is often integrated into healthcare systems to streamline operations and enhance efficiency [7–9]. For instance, studies have shown that OR methodologies are employed to analyze patient flow, optimize scheduling, and improve the allocation of healthcare resources [10, 11]. Conversely, in low- and middle-income countries, the application of OR focuses on overcoming barriers to care, such as transportation, affordability, and the availability of skilled personnel [1–3, 12]. Eye health institutions in these settings often use OR to design outreach programs, optimize the distribution of scarce resources, and monitor the impact of interventions on reducing blindness and visual impairment [13–15]. While OR is a powerful tool for improving eye health services, its application and effectiveness differ markedly between high-income and low- and middle-income countries [1–9]. HICs benefit from established OR frameworks and training programs that enhance healthcare delivery, whereas LMICs face challenges related to resource constraints and the need for capacity building.

Therefore, it was crucial to evaluate the ORCB program’s impact on participants and their institutions before scaling up this approach. More specifically, the primary objective of this evaluation was to assess research competencies, including developing research questions, designing studies, developing protocols, analysing data, and disseminating findings. The secondary objective was to identify personal and institutional factors that enabled or hindered the practice of OR by program participants.

## Materials and methods

### About the program

The Seva Foundation USA and Seva Canada are international non-governmental organisations.

(NGOs) dedicated to improving global eye care. They have long supported research capacity-building initiatives, partnering with the Global Sight Initiative (GSI) [16], a collaborative network of Seva partners working to strengthen eye health systems worldwide. Recognising the critical role of OR, the Seva Foundation USA, Seva Canada and the Public Health Foundation of India (PHFI), through its Indian Institute of Public Health, Hyderabad (IIPH-H) campus, collaborated to conduct the Operational Research and Capacity Building (ORCB) program. This included four selected eye care hospitals, three in Nepal and one in India, from 2019 to 2022 [6]. The program incorporated structured workshops, mentorship, and support in data management, statistical analysis, and presentation of data to strengthen participants’ research competencies [6]. 

It aimed to empower eye care professionals, hospital administrators and staff by equipping them with the knowledge and skills necessary to conduct OR. These included developing research questions, designing studies, creating study protocols, implementing research, analysing data, and disseminating findings through scientific writing. Strengthening research capacity within these institutions contributes to better patient outcomes by optimising screening programs, improving referral pathways, and increasing access to quality eye care. On a broader scale, the program contributes to public health efforts by fostering evidence-based decision-making, promoting equitable access to eyecare, and integrating research-driven solutions into national and regional policies.

However, due to the COVID-19 pandemic, the original plan of in-person workshops was converted into an online model, which was successfully executed by IIPH-H faculty over three years. This success led to a renewed partnership focusing on further strengthening the operational capacity of the initial four partners and expanding support to more eye-care institutions. Knowledge and practices were evaluated 4 months after the training.

### Inclusion criteria

The institutions were selected based on two key criteria. First, the presence of an established data collection system within the institution. Second, the institution’s commitment to the program outcomes, demonstrated by dedicating 4 h weekly to the initiative, indicates serious engagement. Participants were recruited who were (1) affiliated with one of the selected institutions and (2) commitment to completing the program, including attending all the training sessions and contributing to research projects.

### Evaluation design

The evaluation employed a mixed-method design, integrating both quantitative and qualitative data to provide a comprehensive assessment of the ORCB program’s impact.

### Evaluation levels and methods

The evaluation of participants OR knowledge was conducted in several ways: a self-reported questionnaire, interviews and observation with participants by the mentors.

#### First level: self-reported questionnaire

A self-reported questionnaire included three sections was shared with all participants via Google Forms.

1. OR skills before and after the capacity-building initiatives: The questionnaire focused on skills included in the training program, such as formulating research questions, designing studies, developing protocols, implementing research, analyzing data, and disseminating findings. Competency levels before and after the program were assessed using scores measured on a Likert scale from 1 to 5, where higher scores indicated greater levels of competency. Using R Statistical Software, a paired t-test was applied for data values of paired measurement to examine the mean change and standard deviation (SD) in knowledge score, with appropriate 95% confidence intervals (CI). A p-value < 0.05 was considered statistically significant.
2. Practice in the hospital after ORCB: This focused on research-related practice either in designing or developing the protocols and training other staff at their respective hospital. Categorical variables were summarized using frequencies and percentages.
3. Motivating factors/enablers and challenges: To determine the factors that have influenced study participants’ research practice since completing the ORCB program. The mean score was calculated for each factor using a Likert scale ranging from 0 to 9. The highest score indicates better support or improvement in their research practice.

#### Second level: interviews and observation

Assigned mentors conducted face-to-face interviews with program participants, including eye-care professionals, hospital administrators, and staff, while excluding patients from each participating institution. These interviews were recorded with prior permission from the hospital authorities and the interviewees. The qualitative data collected through these interviews provided in-depth insights into the participants’ experiences, challenges, and the institutional impact of the program.

The qualitative data underwent thematic analysis, a method that involved identifying, analyzing, and reporting patterns (themes) within the data to understand participants’ experiences, challenges, and the overall impact of the training program. This was done by two people to ensure consensus. Additionally, the mentors underwent a pre-training workshop to standardize guidance across all participating institutions, ensuring uniformity in mentorship.

## Results

A total of 17 participants were enrolled in the program, with 13 (76.5%) from Nepal and 4 (23.5%) from India. A Google form survey was distributed to all the participants. Of the 17 participants, 15 (88.2%) completed the survey. Response rates varied across hospitals, with two hospitals having 100% participation, while in the other two hospitals, the response rates were 75% (3 out of 4) and 80% (4 out of 5), respectively. Among the participants, 9 (53%) were male, and 10 (59%) were between the ages of 30 and 40. The educational background of the participants included 4 ophthalmologists, and 5 each optometrists and ophthalmic assistants. (Table 1)

**Table 1:** Demographic characteristics of the study participants

Characteristics	Frequency (*n*)	Percentage (% of T)
Gender		
Male	9	53%
Female	8	47%
Age groups (years)		
30–40	10	59%
41–50	7	41%
Educational level		
Ophthalmologists	4	24%
Optometrists	5	29%
Ophthalmic Assistant	5	29%
Others (Hospital Administrators and Program Managers)	3	18%
Response to survey		
Yes	15	88%
No	2	12%
Total (T)	17	100%

*Legend*: *T* Total number of participants

### Program delivery/knowledge/skills/competency (before and after skilling: before and after score comparison):-

#### Quantitative

Responses from the participants suggest that the knowledge level improved significantly in all the domains (Table 2). The knowledge level increased on how to conduct a literature review, how to formulate a research question and knowledge of various study designs. However, topics like sample size calculations, data analysis plans, and manuscript writing required additional skilling.

**Table 2:** Knowledge level improvement before and after the ORCB program using the paired t-test (*n* = 15 participants)

Domains	Knowledge			
	Before Skilling (mean ± SD)	After Skilling (mean ± SD)	Change in mean score (95% CI)	*P*-value
Literature review	2.7 ± 1.1	3.9 ± 0.5	1.2 (0.8, 1.6)	< 0.0001
Formulating research questions	2.6 ± 1.1	3.9 ± 0.7	1.3 (0.9, 1.7)	< 0.001
Sampling techniques	2.4 ± 1.2	3.5 ± 0.8	1.1 (0.5, 1.6)	0.0007
Manuscript writing	2.3 ± 1.2	3.3 ± 0.9	1.0 (0.6, 1.4)	< 0.0001
Study designs	2.2 ± 1.2	3.6 ± 0.8	1.4 (0.9, 1.9)	0.0001
Data collection tools	2.1 ± 1.1	3.6 ± 0.8	1.5 (1.0, 1.9)	< 0.0001
Sample size calculation	1.9 ± 1.0	3.1 ± 0.8	1.1 (0.6, 1.6)	0.0003
Data analysis	1.9 ± 0.7	3.2 ± 0.9	1.3 (0.8, 1.9)	0.0002

*Legend*: *SD* Standard Deviation, *CI* Confidence interval

#### Qualitative

##### Knowledge improvement and publication success

Overall participants felt that their knowledge improved substantially after joining the program. Two ophthalmologists expressed that their understanding improved a lot and that their respective teams could successfully publish two manuscripts in peer-reviewed journals.

##### Effective workshop approach, with room for material improvement

Participants appreciated the workshop’s standard and approach, considering it efficient. However, feedback indicated that workshop materials, while generally good, needed further standardization, especially regarding learning formats. The need for more structured and standardized training modules, particularly for beginners, was emphasized. “Should develop standardized training modules, especially for the beginners.” Another participant felt that some of the material was confusing and expressed concern- “I get even more confused after going through the training content when I train my students”. Her suggestion was to revise the program content though she did not provide any specific suggestions.

##### Need for consistent and long-term mentorship

One participant also mentioned that for their hospital, a frequent change of mentors hampered their work as each mentor differed in their approach which led to confusing signals. This team suggested that the presence of a dedicated mentor for a longer duration might be helpful for them to learn the skills better.

##### Difficulties with online delivery and suggested solutions

Three participants stated that the online delivery of the program had lots of challenges, especially due to internet connectivity issues. They suggested that the provision of lecture transcripts could be an option to overcome such challenges.

##### Statistical learning and mentor support

Two participants mentioned that they partially completed the ORCB program as the in-person statistics workshop was attended by two out of four members. One member expressed unhappiness that though they wanted to attend but were not allowed by the hospital administration. The person requested that “Next time when Seva/IIPH-H organize such an event, they can consider calling all members instead of a few of them.” One person mentioned that learning from the program was minimal as he/she did not learn any additional statistical concepts. Some participants opined that they were not confident in responding to the comments of reviewers on statistics when they submitted a manuscript and had to approach the team mentor from the training institution to respond to the same. Unfortunately, this respondent did not share this concern with the designated team mentor from the training institution as he/she felt demotivated as adequate statistical analytical skills were not picked up.

##### Need for incorporating health economics

While acknowledging the program’s strengths, participants identified challenges, particularly in the areas of cost-effectiveness and health economics. One participant expressed “Despite the strengths, the program faced challenges in topics such as cost-effectiveness and health economics within the hospital.” They suggested that incorporating a health economics component into the projects could address the hospital authorities’ concerns about financial sustainability.

### Practices in the hospital after skilling

#### Quantitative

While 66.7% (*n* = 10) of participants were involved in designing study protocol development, 73.3% (*n* = 11) participants took part in data analysis at their hospital (Either analysis of routine data, research data, or both). Importantly, 60% (*n* = 9) of participants trained other hospital staff or students on topics they learnt during the training program. (Table 3) Most of their training was related to literature search (*n* = 7; 77.8%) formulating research questions and objectives (*n* = 8; 88.9%) and designing tools for data collection (*n* = 5; 55.6%). Most of the training for the hospital students and staff was related to these areas. (Table 4)

**Table 3:** Research-related practice among participants after the ORCB program. Research practice Post-Training (*n* = 15 participants)

Research-related practice	Yes *n* (% of T)	No/Not planned *n* (% of T)	Planned, *n* (% of T)
Designed any study or developed protocol	10(66.7%)	2 (13.3%)	3(20%)
Performed data analysis	11(73.3%)	1 (6.7%)	3(20%)
Trained any hospital staff or students	9(60%)	2 (13.3%)	4(26.7%)
Participated in manuscript writing	10(66.7%)	2 (13.3%)	3 (20%)

**Table 4:** Research-related practice among participants after the ORCB program. Specific contribution in protocol development & training

Contribution type	Protocol Development (T = 10) *n* (% of T)	Training (T = 9), *n* (% of T)
Literature search	5(50%)	7(77.8%)
Formulating the research question and objectives	8(80%)	8(88.9%)
Identifying epidemiological study design	4(40%)	3(33.3%)
Designing tools for data collection	7(70%)	5(55.6%)
Sample size calculation/Sampling technique	4(40%)	3(33.3%)
Data analysis plan	4(40%)	4(44.4%)

#### Qualitative

##### Positive impact on hospital services and operational practices

The overall improvement of the hospital services had been promising after the training. One participant expressed that “Significant changes have been observed in the research and operation aspects.” The approach to work now involves a combination of learning, research, and operational activities. The software for electronic hospital records was updated to facilitate seamless integration with routine work. Also, efforts have been made to improve the connections between the patients from vision centres and base hospitals based on the learning from the training program.

##### Potential for self-learning within hospitals

Another participant from the same team who works in the clinical lab felt that there was tremendous scope for self-learning at the hospital as a large amount of data was available in the different departments of the hospital. “One participant mentioned having improved a lot in designing studies and manuscript writing based on the fundamental principles learnt from the training program”.

##### Internal training initiatives

Nine participants took the initiative to train additional teams within the hospital. This internal training covered topics such as proposal writing, data management, analysis, and scientific writing, indicating a positive ripple effect of the original training program.

##### Necessity of building a dedicated research team

Participants emphasized the importance of research in improving hospital services. However, they noted that research efforts were often driven by individual clinicians rather than a coordinated team. One participant expressed - “Building a team of researchers who constantly work on the research flow and its outcome is critical rather than only clinicians spending time as they are quite busy. Unfortunately, it is all about the individual approach and not a team approach.”

##### Insufficient time for implementation

One participant expressed a concern that -“The short time for implementation adds pressure, impacting the program’s overall effectiveness”. This highlights the need for better time management.

##### Gaps in research dissemination and accountability

Despite the progress in research activities, one participant informed that there is a lack of formal systems for disseminating research findings within the hospital. Although verbal updates were provided to hospital authorities 3–4 times a year, there is no provision for documented formal meetings to keep track of the action plans. Also, when researchers at our hospital publish any paper, there is no system to disseminate the findings among the other stakeholders.

### Motivators and challenges

#### Quantitatiive (*n* = 15)

**Fig. 1:**
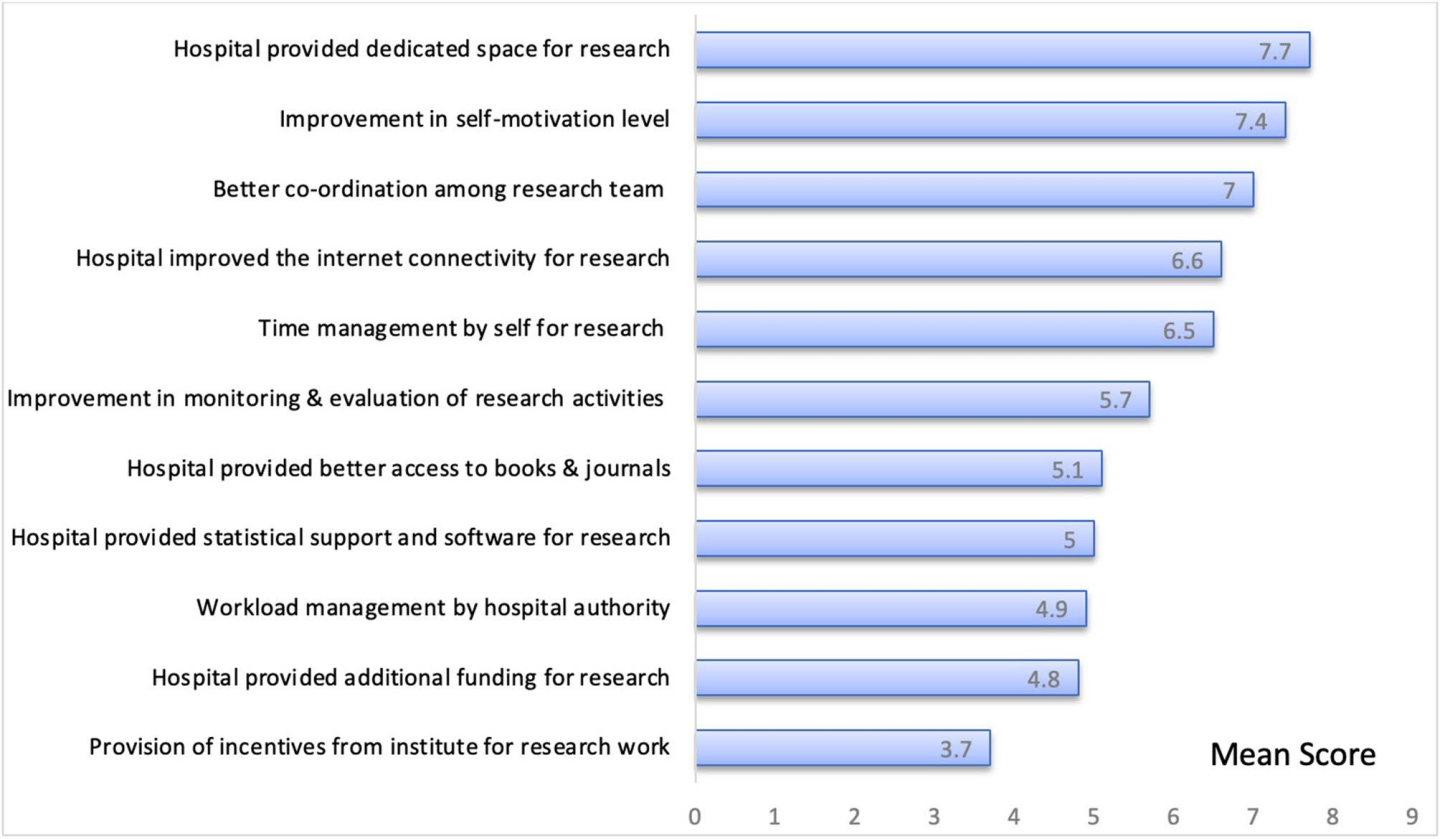
Mean score of motivating factors/enablers and challenges among participants

Figure 1: Motivating factors/enablers and challenges faced by participants. This figure presents the quantitative analysis of the primary motivators and challenges reported by participants in the ORCB program. Notably, the highest scores reflect significant improvements in factors that contribute to effective research practices. Major challenges identified include the absence of institutional incentives, additional funding for research provided by the hospital, workload management by the hospital authority and no provision of statistical software for analysis. Conversely, self-motivation, coordination among the research team and time management by self were the major enablers.

#### Qualitative

##### Need for enhanced statistical tools and software

Participants discussed the importance of using robust statistical software for data analysis. One participant opined “Increase the use of statistical tools like Stata software for more robust analysis.” One participant expressed he/she was directly involved in guiding students’ (postgraduates) research. “I guide 3–4 postgraduates every year as a part of my job and I guide them for their thesis work. So, it is a routine process for me”. Two other participants mentioned that they had received a few of the thesis protocols from the students for review. Both were guiding optometrists for their thesis. Lack of a licensed version of the software at the hospitals also demotivates them- “We learned the basics during the training, but because we do not have licensed software for data analysis like SPSS or Stata software, we are handicapped in doing that. The hospital administration can help us in getting that.”

##### Balancing clinical work and research

Balancing clinical work and research was identified as a major challenge, particularly for clinicians. “Conducting research and clinical work is a double burden for us when management wants us to work on research without reducing our clinical workload”.

##### Lack of incentive for research

For clinicians, motivators like research work will add to their resumes and help in promotion. Nothing much of an incentive is there for the research work.

## Discussion

Implementing an ORCB program, in resource-poor areas of India and Nepal, during the COVID pandemic required patience, persistence and a very high level of mutual trust, tolerance and cooperation. Despite the obvious challenges, the program successfully enhanced institutional and individual interest in and knowledge of research and showed its critical importance in optimizing the effective and efficient use of very limited healthcare resources.

The limited number of health human resources, minimal research capacity, and constrained healthcare systems in low-income settings are well described [1–9]. The contrasts with high-income countries, with robust healthcare infrastructure and extensive research resources, make existing literature on ORCB development, in resource-rich settings, essentially irrelevant to the settings described herein [1–9]. What is clear, is that the potential for operational research impact, in resource-poor settings, greatly exceeds that of rich settings.

Strengthening OR capacity in primary healthcare (PHC) is particularly crucial given the ongoing efforts to reform and improve PHC services in LMICs. Recent initiatives, such as India’s Health and Wellness Centres (HWCs) under the Ayushman Bharat program, emphasise comprehensive primary care, community-level service delivery, and evidence-based decision-making [17]. By embedding research capacity within primary healthcare systems, ORCB programs can help translate evidence into policy and practice, ultimately improving service efficiency and health outcomes. For example, a recent study from India evaluating the impact of providing cataract surgeries at a rural primary healthcare centre demonstrated how strategic interventions can bridge the gap in eye care accessibility, especially for underserved populations [18]. This highlights the importance of embedding research capacity-building within the eyecare programs to facilitate evidence-based service delivery.

In a post-hoc survey, ORCB participants in all 4 settings reported improvement in their ability to conduct literature reviews, formulate research questions, design studies, develop data collection tools and conduct data analysis. The observed improvements align with other capacity-building efforts in healthcare, such as the Primary Eye Care training program in Tanzania [19] and the research capacity-building initiative for non-communicable diseases in India, where participants similarly reported increased confidence and competence in conducting research activities [20]. Another study from Rwanda focused on training nurses in primary eye care examinations, which showed improvement in knowledge and building capacity, hence strengthening the eye health care system in their country [21]. 

In the survey, ORCB participants, noted several areas needing further attention, particularly statistical methods and manuscript writing, both of which will require deeper engagement and ongoing support. This finding is consistent with other research training programs, where continued mentorship and advanced workshops have been shown necessary to reinforce research methodology acquisition [6, 7, 22–24]. Additionally, a shift from in-person workshops to an online model brought both opportunities and challenges, particularly in maintaining participant engagement and ensuring effective mentorship. A study comparing online versus in-person interviews for qualitative studies has highlighted key feasibility and ethical considerations, including issues of rapport-building and digital accessibility, which were also relevant in this program [25]. Interviews and discussions with hospital staff support the survey findings, with almost all participants acknowledging substantial improvements in their research skills. However, they made several suggestions for improvement. They noted that the frequent change of mentors led to confusion, as each mentor had different approaches to research guidance. As noted elsewhere, a structured mentorship program, with clear communication channels and dedicated mentors for extended periods, is necessary for participants to fully develop their research skills [22–24, 26–28]. Furthermore, participants suggested that training materials should be standardized and made clearer, particularly for beginners. More structured modules with clear, step-by-step guidelines would also help participants to both apply and teach the material.

Most participants, particularly clinicians, noted that their institutions did not fund their time for research activities. This problem is common in LMICs, where healthcare professionals often face heavy clinical workloads, limiting their ability to dedicate time to research [29–31]. In addition, the institutions did not fund basic research tools, such as licensed statistical software, hindering their ability to analyse data.

## Conclusions

The ORCB program succeeded in its short-term goal of engaging individuals and institutions in research-related activities and started to build, what is described elsewhere as a culture of research within the participating hospitals [32–34]. However, to date, research efforts remained largely driven by individuals, rather than a coordinated institutional approach. Nevertheless, three of the four partner programs did begin to establish dedicated research teams.

Future ORCB program activities include not only continuing to build research capacity but also using the Plan–Do–Study–Act tool, a structured, iterative method used for continuous improvement in healthcare and research [35, 36], to mentor the use of operational research findings to build evidence-based administrative decision-making structures and to improve the quality of administrative data infrastructure. Application of research findings is the obvious next step to improving the quality and efficiency of eye care services in these regions.

## Limitations

The study had limitations, particularly the small number of eye care institutions and the specific contexts of India and Nepal. The COVID pandemic also affected the program’s delivery, eliminated face-to-face mentoring of research activities and limited capacity to assess institutional administrative structures and decision-making processes.

## Supplementary Information

Supplementary Material 1.

Supplementary Material 2.

Supplementary Material 3.

## Data Availability

The datasets used and/or analysed during the current study are available from the corresponding author on reasonable request. The materials have been uploaded to the supplementary file.

## Abbreviations

OR: Operational Research
ORCB: Operational Research and Capacity Building
PHFI: Public Health Foundation of India
IIPH-H: Indian Institute of Public Health, Hyderabad
HICs: High-Income Countries
LMICs: Low- and Middle-Income Countries
SD: Standard Deviation
CI: Confidence Interval
GSI: Global Sight Initiatives
NGOs: Non-Governmental Organisations
HWCs: Health and Wellness Centres
PHC: Primary Health Care
