# Supplementary material for: Using operational research as a tool to improve eye health services and systems in low-and middle-income settings: lessons from India and Nepal
Source: BMC Med Educ. 2025 Aug 26;25:1202. doi: 10.1186/s12909-025-07803-6 (PMC12379328; doi:10.1186/s12909-025-07803-6)
Supplement: Supplementary file 1 — Supplementary Material 1. [file 12909_2025_7803_MOESM1_ESM.pdf]

ORCB Program - Interview guide

Program delivery:

- How would you assess your overall experience with the ORCB program?
- In what ways, if any, did your knowledge and skills improved as a result of the program?
- How would you rate the quality and clarity of the workshop materials provided?
- Can you provide some suggestions where the training materials or delivery could be enhanced?
- How would you describe the support provided by your mentor(s)?
- Did frequent changes in mentors affect your learning experience?
- Did you face any challenges during the program (e.g., online delivery)?
- Did you feel the training met your expectations in terms of improving your statistical knowledge?
- Do you think the program should cover certain topics?

Practices in the hospital after skilling:

- How has your hospital benefited from the training program in terms of services and operations?
- What aspects of the training program facilitated self-learning in your practice?
- Does your hospital encourage or provide resources for self-learning activities?
- Have you implemented any internal training sessions at your hospital? If so, What topics have been covered?
- Who is responsible for managing research activities within your hospital?
- Do you feel that the allocated time was insufficient for the implementation of the program?
- How are research findings shared with the hospital authorities and other stakeholders?

Motivators and Challenges:

- How do you handle data analysis for your research or for training at your hospital? Which specific software is used for analysis?
- How do you balance your time between clinical work and conducting research?
- What motivates you to engage in research activities alongside your clinical responsibilities?
