# Supplementary material for: Using operational research as a tool to improve eye health services and systems in low-and middle-income settings: lessons from India and Nepal
Source: BMC Med Educ. 2025 Aug 26;25:1202. doi: 10.1186/s12909-025-07803-6 (PMC12379328; doi:10.1186/s12909-025-07803-6)
Supplement: Supplementary file 2 — Supplementary Material 2. [file 12909_2025_7803_MOESM2_ESM.pdf]

ORCB Program – Self Reported Questionnaire

This form is for the participants who participated in the ORCB-1 program designed and delivered jointly by Seva Foundation, Seva Canada and IIPH-H.

The participants must respond to the questions INDIVIDUALLY and *NOT in a group*.Your response will be completely anonymous.

*Indicates a required question

1. Rate your competency for the below-mentioned items ***before you joined the ORCB program***. Response 5 indicates the highest level of competency, whereas 1 indicates the lowest level of competency. *

   Provide a single rating for each.

**Domains**	**5**	**4**	**3**	**2**	**1**
Literature review					
Formulating research questions					
Sampling techniques					
Manuscript writing					
Study designs					
Data collection tools					
Sample size calculation					
Data analysis					

2. Rate your competency for the below-mentioned items ***after you completed the ORCB program***. Response 5 indicates the highest level of competency, whereas 1 indicates the lowest level of competency. *

Provide a single rating for each.

**Domains**	**5**	**4**	**3**	**2**	**1**
Literature review					
Formulating research questions					
Sampling techniques					
Manuscript writing					
Study designs					
Data collection tools					
Sample size calculation					
Data analysis					

3. Did you contribute to designing any study or protocol development **after the ORCB program?** *

Mark only one oval.

Yes

No/ Not planned

Planned

4. If your answer is 'yes' to the previous question, which part of the research did you contribute to?(Choose all that apply). ***Leave it blank if your answer is not 'yes'.***

Select all that apply.

**Contribution type**	(✓)
Literature search	
Formulating the research question and objectives	
Identifying epidemiological study design	
Designing tools for data collection	
Sample size calculation/ Sampling technique	
Data analysis plan	

5. Did you do any data analysis at your hospital ***after the ORCB program?*** *

Mark only one oval.

- Yes
- No/ Not planned
- Planned

6. Have you trained any hospital staff or students ***after completing the ORCB program?*** *

Mark only one oval.

- Yes
- No/ Not planned
- Planned

7. If your answer is 'yes' to the previous question, which of the following training you have imparted**?** (Choose all that apply) ***Leave it blank if your answer is not 'yes'.***

Select all that apply.

**Contribution type**	(✓)
Literature search	
Formulating the research question and objectives	
Identifying epidemiological study design	
Designing tools for data collection	
Sample size calculation/ Sampling technique	
Data analysis plan	

8. Did you take part in any manuscript writing ***after the ORCB program***? *

Mark only one oval.

- Yes
- No/ Not planned
- Planned

9. The following question identifies areas that may serve as either motivating factors/enablers and challenges depending upon the context and individual perception. We want to know if these factors influenced your research practice since you have completed the ORCB program.

Provide a single score for each between 0-9, where '0' represents the lowest score of support or improvement in research practices and '9' indicates the highest. *

**Motivating factors/enablers and challenges**	**Score (0-9)**
Hospital provided dedicated space for research	
Improvements in self-motivation level	
Better co-ordination among research team	
Hospital improved the internet connectivity for research	
Time management by self for research	
Improvement in monitoring & evaluation of research activities	
Hospital provided better access to books & journals	
Hospital provided statistical support and support for research	
Workload management by hospital authority	
Hospital provided additional funding for research	
Provision of incentives from institute for research work	
