# Supplementary material for: Using operational research as a tool to improve eye health services and systems in low-and middle-income settings: lessons from India and Nepal
Source: BMC Med Educ. 2025 Aug 26;25:1202. doi: 10.1186/s12909-025-07803-6 (PMC12379328; doi:10.1186/s12909-025-07803-6)
Supplement: Supplementary file 3 — Supplementary Material 3. [file 12909_2025_7803_MOESM3_ESM.pdf]

Institutional Ethics Committee

Indian Institute of Public Health-Hyderabad /
Public Health Foundation of India

Communication of Decision of the IEC[1]

Form II

**TRC-IEC No Application No:**	IIPHH/TRCIEC/406/2024	**Date:**		**14-11-2024**	
**Project Title:**	Operational Research in Capacity Building (ORCB) for eyecare hospitals in India and Nepal and related evaluation programs				
**Principal Investigator:**	**Dr. GVS Murthy**				
**Review**	**Full Review**	☐		**Expedited Review**	☐
	**Waiver**	☒			
**Date of review:**					
**Date of previous review:**	***(in case of re-submitted applications)***				
**Decision of the IEC:**	**Approval**	☒		**Resubmission**	☐
	**Conditional Approval**	**Study can begin**	☐	**Study cannot begin**	☐
**Requirements to be fulfilled in case of conditional approval:**					
**Suggested alterations in case of resubmission:**					
**In case of approval, *recommended for a period of :***	**Approval is valid for a period of one year from the date of issue**				
**Comments:**					

Please note: Beginning of the research based on this approval implies acceptance of the following conditions:

1. *PI will inform the Secretariat of the start date of the study.*
2. *The PI will inform the IEC in case of any adverse events.*
3. *The PI will inform the TRC (Technical Review Committee) and IEC in case of any change of study procedure (including- changes in the informed consent form, recruitment procedure, potential research participant information), site and investigator.*
4. *The PI will inform the TRC - IEC Secretariat on termination of the study and submit a final report within 3 months of completion of the study.*
5. *Members of the IEC have the right to monitor the study with prior intimation.*
6. *Progress report to be submitted to the TRC-IEC Secretariat every 6 months from the date of start of study.*
7. *This permission is only for the period mentioned above.*

Sirshendu Chaudhuri (signature) | (signature)

Name and signature of Member Secretary | **Chairperson, IEC, IIPH**

[1] Adapted from the ICMR form: available at http://www.icmr.nic.in/bioethics/Communication%20of%20Decision%20of%20the%20IEC.doc

Dr. Sirshendu Chaudhuri **Dr. Vijay Yeldandi,**
